# Multimodal super-resolution optical microscopy visualizes the close connection between membrane and the cytoskeleton in liver sinusoidal endothelial cell fenestrations

**DOI:** 10.1038/srep16279

**Published:** 2015-11-09

**Authors:** Viola Mönkemöller, Cristina Øie, Wolfgang Hübner, Thomas Huser, Peter McCourt

**Affiliations:** 1 Biomolecular Photonics, Department of Physics, Bielefeld University, Universitätsstr. 25, 33615 Bielefeld, Germany; 2 Faculty of Health Sciences, Department of Medical Biology, Vascular Biology Research Group, The Arctic University of Norway, 9037 Tromsø, Norway; 3 Department of Internal Medicine, and NSF Center for Biophotonics, University of California, Davis, 2700 Stockton Blvd., Ste. 1400, Sacramento, CA 95817, USA

## Abstract

Liver sinusoidal endothelial cells (LSECs) act as a filter between blood and the hepatocytes. LSECs are highly fenestrated cells; they contain transcellular pores with diameters between 50 to 200 nm. The small sizes of the fenestrae have so far prohibited any functional analysis with standard and advanced light microscopy techniques. Only the advent of super-resolution optical fluorescence microscopy now permits the recording of such small cellular structures. Here, we demonstrate the complementary use of two different super-resolution optical microscopy modalities, 3D structured illumination microscopy (3D-SIM) and single molecule localization microscopy in a common optical platform to obtain new insights into the association between the cytoskeleton and the plasma membrane that supports the formation of fenestrations. We applied 3D-SIM to multi-color stained LSECs to acquire highly resolved overviews of large sample areas. We then further increased the spatial resolution for imaging fenestrations by single molecule localization microscopy applied to select small locations of interest in the same sample on the same microscope setup. We optimized the use of fluorescent membrane stains for these imaging conditions. The combination of these techniques offers a unique opportunity to significantly improve studies of subcellular ultrastructures such as LSEC fenestrations.

As super-resolution optical microscopies are beginning to mature and are demonstrating their value for live cell imaging applications, a number of technical issues are becoming quite apparent^1^,^2^,^3^,^4^,^5^,^6^. In particular, depending on the specific super-resolution optical modality available, certain trade-offs with respect to sample preparation or the use of fluorescent stains need to be made. Some techniques, such as single molecule localization microscopy, provide very high spatial resolution but require rather long overall signal accumulation times by collecting several thousands of frames to achieve their high resolution. Other techniques, e.g. three-dimensional structured illumination microscopy (3D-SIM) and derivatives thereof, are fast or can be utilized in a fast acquisition mode^7^,^8^,^9^, but this is often bought at a higher, but still limited spatial resolution. Most applications, however, in particular live cell imaging applications require both, namely a rapid search mode that enables the identification of cells/areas of interest, which can then be followed by more time-consuming, higher resolution microscopy of the relevant features.

One way by which such a search mode can be achieved is using a conventional fluorescence microscopy platform in combination with an integrated super-resolution modality. Here, the conventional mode is used to rapidly survey a sample, to identify locations of interest, and to then revisit them with super-resolution microscopy. If, however, the targets of interest in the study are only present at dimensions well below the optical diffraction limit, then a combination of super-resolution techniques is needed, where one modality provides the ability to rapidly survey the sample, ideally in multiple color channels, which can then easily be switched to an even higher resolving technique if needed. Cellular fenestrations in liver sinusoidal endothelial cells (LSEC) present such a challenging target. The size of fenestrations, i.e. trans-cytoplasmic pores with a diameter between 50–200 nm, is well below the optical resolution limit, making them impossible to see with conventional optical microscopy^10^,^11^. LSECs are specialized endothelial cells lining the blood vessels (sinusoids) that permeate the liver. These cells contain a large number of such fenestrations, which in essence act as molecular sieves between the blood and the underlying hepatocytes. LSECs retain blood cells in the vessels, while molecules, such as metabolites, plasma proteins, pharmaceutical drugs, lipoproteins, and smaller viruses (<200 nm) can pass this barrier^10^. Despite their physiological importance, very little is known about the dynamics and function of fenestrations in LSECs because of their small size and a lack of specific markers^12^. These structures are typically arranged in “sieve plates”, i.e. assemblies of fenestrations in regions where LSECs are extremely thin (approx. 300 nm on average) and where neighboring fenestrations are believed to be separated by actin fibers^13^. 3D-SIM has recently been used to image fenestrations in sieve plates in fixed LSECs and to correlate them with other membrane structures, such as membrane proteins and lipid rafts^14^,^15^. More recently, single molecule localization microscopy, i.e. *direct* stochastic optical reconstruction microscopy (*d*STORM) was used to image fenestrations at even higher optical resolution^16^. The combination of both, however, in order to take advantage of the specific strengths of each technique, has not yet been utilized.

Few attempts at multi-modal super-resolution microscopy have been demonstrated so far. Matsuda *et al*. used a 3D-SIM optical platform to acquire data for single molecule localization microscopy^17^. Rossberger *et al*. combined SIM and single molecule localization microscopy into a single platform and demonstrated this by imaging identical regions of H3K293 cells^18^. Most recently, Hamel *et al*. used 3D-SIM to obtain multi-color SIM data and compare them with the higher resolution obtained by single molecule localization microscopy^19^.

Here, we demonstrate the ability of a single optical platform to rapidly search, survey, and stitch large sample areas for relevant feature sizes in multi-color operation on freshly isolated fixed rat LSECs. Once areas with sieve plates are identified, the illumination and acquisition mode of the microscope is switched to *d*STORM to revisit these locations and enable the acquisition of images of the LSEC cytoskeleton and fenestrations with up to 20 nm optical resolution. The combination of both methods enable us to also directly compare areas imaged by two different super-resolution modalities, which ultimately enables us to reveal the close association between the LSEC cytoskeleton and the plasma membrane.

## Results

**Figure 1:**
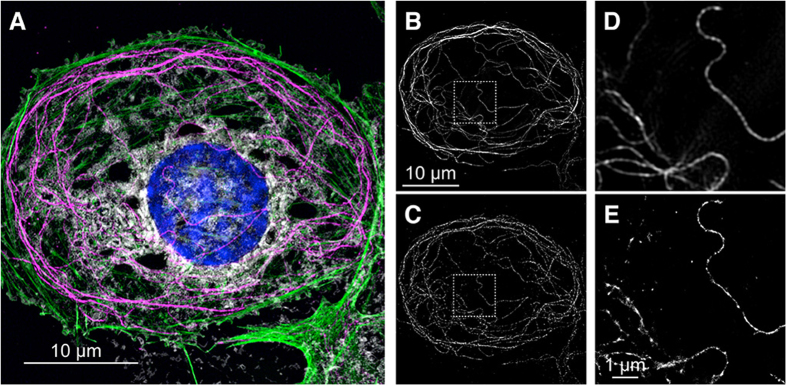
Correlating 3D-SIM and *d*STORM images of a rat liver sinusoidal endothelial cell (LSEC). (**A**) Maximum intensity z-projection 3D-SIM image of a 4-color-stained fixed rat LSEC. The nucleus was stained with DAPI (blue), actin filaments with Phalloidin-Alexa488 (green), membranes with CellMask Orange (white), and tubulin structures with anti β-tubulin mouse antibody followed by an anti-mouse IgG-Alexa647 antibody (magenta). The maximum intensity z-projection corresponds to a sample thickness of 750 nm. (**B**) Maximum intensity z-projection 3D-SIM image of the tubulin channel from (A) compared to the *d*STORM reconstruction (**C**) of the same cell. (**D**) Enlarged 3D-SIM and (**E**) *d*STORM images of the ROIs (dashed-line boxes) shown in (**B,C**). The *d*STORM image shows a direct correlation with the corresponding 3D-SIM image, but with an optical resolution of approx. 20 nm. Note that the *d*STORM image is obtained in HiLo mode, where in thicker parts of the cell not all of the entire volume of the cell is illuminated, resulting in small differences between the images. The single frame exposure time of the *d*STORM image was 20 ms and a total of 10000 frames were used for the reconstruction. The sample was mounted in Vectashield.

To evaluate the ability of our setup to perform 3D-SIM and *d*STORM imaging of multiply stained samples, we stained fixed rat LSECs with 4 different fluorophores. We stained the nucleus with DAPI, actin filaments with Phalloidin-Alexa488, membranes with CellMask Orange and microtubules with an anti β-tubulin antibody visualized by using a secondary antibody coupled to Alexa647. This fluorophore is well suited for *d*STORM imaging and it was chosen for single molecule localization microscopy on an existing commercial 3D-SIM platform as it did not require the addition of specific hardware modules^16^,^20^,^21^,^22^,^23^. This is further facilitated by the 642 nm diode laser in our setup providing the highest intensity compared to the other available lasers (300 mW vs 100 mW). High excitation intensities are a prerequisite for successful *d*STORM image reconstruction. Fortuitously, the mounting medium “Vectashield” (Vector Labs) which is commonly used in 3D-SIM applications, enables excellent blinking behavior of Alexa647^24^. The outcome of our 3D-SIM followed by *d*STORM imaging experiment when applied to the tubulin cytoskeleton in LSECs is shown in Fig. 1.

Figure 1A shows the 4-color maximum intensity projection of a 3D-SIM reconstruction of a 750 nm thick z stack of an entire LSEC. The membrane stain (white) delineates the plasma membrane of the cell. Actin filaments (green) are evident as networks throughout the cytosol and at the cell’s circumference. Microtubules, here visualized by the staining of tubulin (magenta), are arranged in nicely separated filaments spanning the cell. The nucleus was stained with DAPI (blue). To better visualize the tubulin network we isolated the color channel in which the fluorescence of the stained tubulin fibers was collected as recorded by 3D-SIM (Fig. 1B) and the same image as recorded by *d*STORM (Fig. 1C). As will be explained in detail further below, the *d*STORM image is obtained in a selective plane-type illumination mode by projecting a highly inclined laminated optical sheet (HiLo) into the sample^25^. Consequently, in thicker parts of the cell not all of the entire volume of the cell is subject to fluorescence excitation in *d*STORM mode. To better compare the ability of both super-resolution microscopy modes to precisely register the same lateral sample location and to compare their respective spatial resolutions, we also show an enlarged overlay of the areas highlighted by dashed-line boxes in these images (Fig. 1D,E). It should be noted that compared to most *d*STORM images that are shown in the literature and which are collected by total internal reflection-excited fluorescence (TIRF), the *d*STORM images shown here are obtained in ringTIRF illumination mode. RingTIRF continuously excites the sample from all directions to ensure a more even illumination of the sample, which further improves the performance of this system for single molecule localization analysis. Also, please note that none of the *d*STORM images shown in this paper used true TIRF illumination, where the illumination arrives at the sample at an angle beyond the angle of total internal reflection. Instead, an angle just below the angle of total internal reflection was chosen to create HiLo illumination.

**Figure 2:**
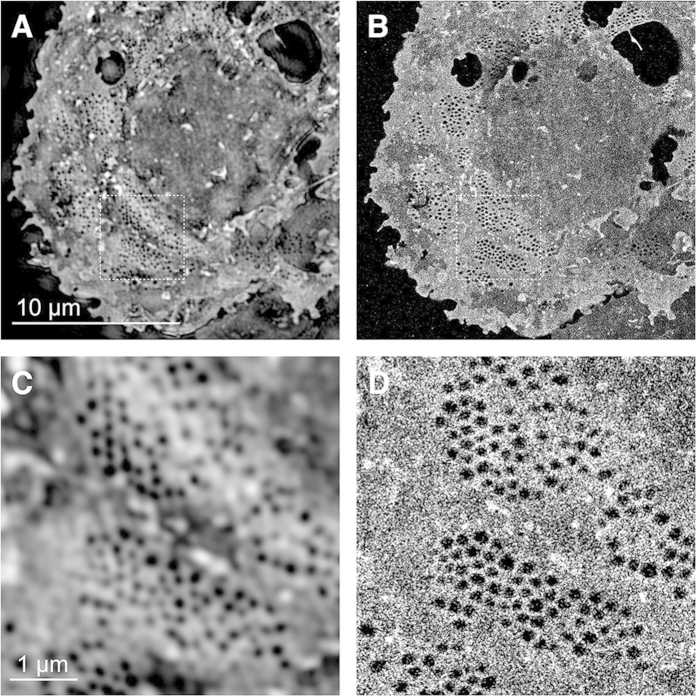
Comparison of 3D-SIM and *d*STORM images of fenestrations in rat LSECs stained with Vybrant DiD. (**A,C**) 3D-SIM and corresponding (**B,D**) *d*STORM images of a fixed rat LSECs stained with Vybrant DiD. The cells were mounted in the reducing buffer OSS + MEA (see Materials and Methods). (**C,D**) show enlarged views of the corresponding ROIs shown in (**A,B**). The 3D-SIM image is a maximum intensity z-projection image of a 500 nm thick part of the cell. The exposure time for a single *d*STORM frame was 20 ms and 15000 frames were processed to reconstruct the images shown in (**B,D**).

Because of the excellent staining of sieve plates by the fluorescent stain Vybrant DiD (see Suppl. Fig. S1) we next evaluated its potential for multimodal imaging by 3D-SIM and *d*STORM. This is shown in Fig. 2, where an entire LSEC was first imaged by 3D-SIM (Fig. 2A,C), and then by *d*STORM (Fig. 2B,D). Figure 2C,D show enlarged views of the areas highlighted in Fig. 2A,B. The significantly improved spatial resolution of the fenestrations by *d*STORM can easily be seen by this direct comparison. It should, however, again be noted that the 3D-SIM reconstructions are obtained after maximum intensity z-projection of the entire cell, whereas the *d*STORM image picks a specific vertical plane within the cell due to the HiLo excitation scheme and is therefore not obstructed by contributions from other vertical planes within the sample.

**Figure 3:**
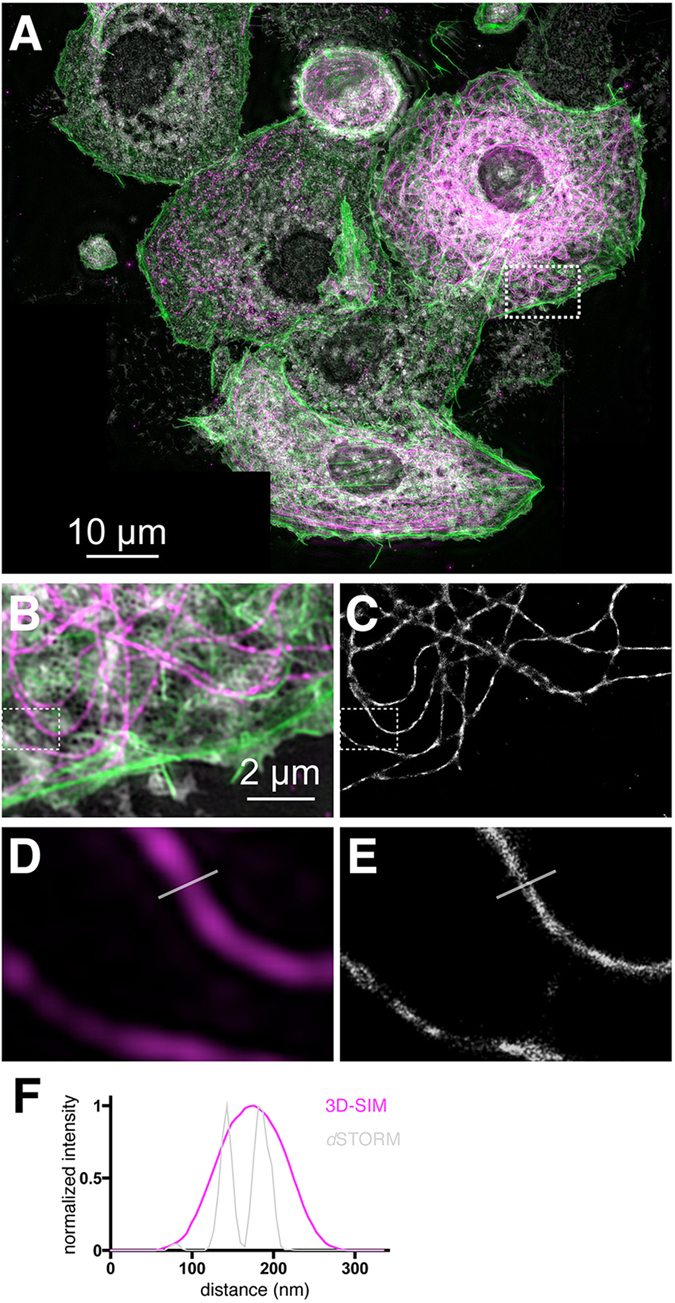
Stitching of multicolor 3D-SIM super-resolution images aids in the search for sieve plates in LSECs. (**A**) Seven multi-color maximum intensity z-projection 3D-SIM images of LSECs were stitched together to produce a large-scale overview image. The fixed rat LSECs were stained for membranes (CellMask Orange, white), actin (Phalloidin-Alexa488, green) and tubulin (mouse anti-β-tubulin and anti-mouse-Alexa647 antibodies, magenta). Scale bar: 10 μm.(**B**) Enlarged view of the ROI shown in (**A**). Tubulin is shown in magenta. (**C**) Corresponding *d*STORM reconstruction of the tubulin network. (**D**) Zoomed view of the β-tubulin channel (magenta) outlined in the 3D-SIM image in (**B**). (**E**) Corresponding *d*STORM image of β-tubulin. The line indicating the location of the cross-section has a length of 330 nm. (**F**) The line sections of the 3D-SIM and *d*STORM tubulin channels show the resolution enhancement achieved by *d*STORM (green line) compared to 3D-SIM (magenta line). The exposure time for a single *d*STORM frame was 20 ms. 10000 frames were used for the reconstruction.

To demonstrate the ability of 3D-SIM to rapidly create a large super-resolved overview of a sample, Fig. 3A shows seven 3D-SIM images of several multi-color labeled LSECs, stitched together using Fiji^26^ to provide an overview of all cells within a specific sample location (see Suppl. Fig. S2A for the stiching pattern of this image). This is particularly important because 3D-SIM is limited in its maximum field of view by the objective lens that is used to create the illumination pattern at the sample. Moving sample locations by approx. 40 μm laterally enables us to rapidly acquire images with a small overlap that can then be stitched together to a single overview image. To present the stitched images as a homogeneous single image, areas that were not imaged in this mode were replaced by a black background. For comparison, Fig. S2 shows the same image with the individual 3D-SIM images outlined by dashed lines. The final image is then used to identify areas of interest for high-resolution *d*STORM imaging, which is considerably more time-consuming than 3D-SIM. Here, our specific area of interest, i.e. a collection of sieve plates, is outlined by the dashed box in Fig. 3A. To permit a direct comparison, Fig. 3B shows an enlarged view of the region of interest in the 3D-SIM image, while Fig. 3C shows the subsequently acquired *d*STORM image. Figure 3D shows an even smaller part of the tubulin channel in the 3D-SIM image, compared to the same region imaged by *d*STORM (Fig. 3E). Line sections in these images are identified and shown in Fig. 3F. As becomes apparent by this direct comparison, *d*STORM reproduces the tubulin fibers with much finer structural detail owing to its high spatial resolution. Of particular note is that in some locations *d*STORM reproduces the tubulin fibers as double-track structures of about 40 nm width (see Fig. 3E), because the thin illumination sheet used for *d*STORM excitation more efficiently excites the fluorophores attached to either side of tubulin by the primary-secondary antibody system rather than the fluorophores that are attached along the vertical direction^27^,^28^.

**Figure 4:**
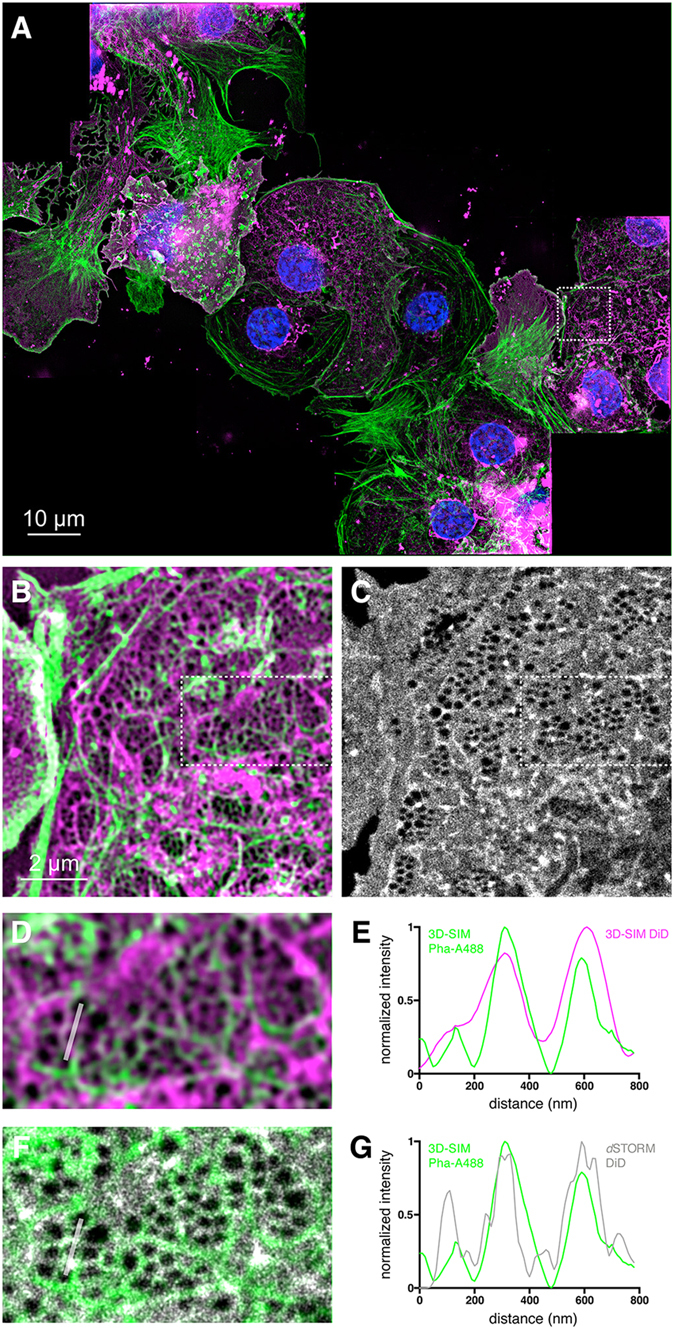
Comparison of cellular features imaged by different super-resolution microscopy modalities. (**A**) Nine multi-color maximum intensity z-projection 3D-SIM images of fixed rat LSECs were stitched together to produce this overview image. The cells were stained for nuclei (DAPI, blue), actin (Phalloidin-Alexa488, green) and membrane (DiD, magenta). (**B**) Enlarged 3D-SIM view of the ROI shown in (**A**) highlighting how fenestrations are surrounded by actin fibers. (**C**) is the corresponding *d*STORM image of the DiD membrane channel. (**D**) is an enlarged view of the ROI shown in (**B**). (**E**) Plot of the line section shown in (**D**) comparing the actin (green) and membrane (magenta) channels of the 3D-SIM image. (**F**) Overlay of the actin channel from 3D-SIM (**D**) shown in green and the membrane channel of *d*STORM ((**C**), outlined box) shown in grey. (**G**) Plot of the line section shown in (**F**) comparing the 3D-SIM actin (green) and *d*STORM membrane (grey) channels. (**G**) The actin line (green) shows the same trend as the membrane *d*STORM line (grey), which suggests that actin filaments support fenestrations.

To determine whether there is a connection between the actin cytoskeleton and the plasma membrane of the fenestrations we directly compared super-resolution fluorescence images collected by both super-resolution microscopy modalities (Fig. 4). Figure 4A is a large multi-color image of several stitched 3D-SIM measurements with a total size of 125 μm × 105 μm (see Suppl. Fig. S2B for the stiching pattern of this image). The nuclei (DAPI, blue), actin filaments (Phalloidin-Alexa488, green) and the plasma membrane (Vybrant DiD, magenta) are shown in this overview. Figure 4B shows an enlargement of the region of interest (ROI) highlighted in Fig. 4A, representing two LSECs in close contact. Fenestrations are arranged in sieve plates as visualized by the membrane stain in the 3D-SIM image. The actin filament is highly resolved and appears to surround the fenestrations. The same ROI was also imaged by *d*STORM (Fig. 4C). A further enlarged part of the 3D-SIM image is shown in Fig. 4D. Figure 4F depicts a direct overlay of the actin channel of the 3D-SIM image in Fig. 4D with the corresponding *d*STORM image. The line sections in Fig. 4E,G compare the actin and membrane channels of just 3D-SIM, and 3D-SIM with dSTORM, respectively. Actin co-localizes well with the membrane stain as seen by *d*STORM, while the 3D-SIM membrane signal lacks spatial resolution. The 3D nature of the 3D-SIM data can be seen more clearly in Suppl. Movie 1.

## Discussion

By extending the capabilities of a commercial 3D-SIM setup (DeltaVision|OMX v4 BLAZE, GE Healthcare, Amersham, UK) we were able to apply two different, but complementary super-resolution techniques, 3D-SIM and *d*STORM, to the same sample for comparative analysis of LSEC ultrastructures. This was accomplished without any physical modifications to the “off the shelf” microscope. *d*STORM image acquisition was performed using TIRF/HiLo excitation as it is typically conducted on custom-built STORM/PALM setups. A particular challenge is the fact that single molecule based localization microscopy becomes very difficult to implement using the low laser power provided by the commercial platform resulting in a laser power density at the sample of less than 0.5 kW/cm^2^. This is approximately two fold less than what is typically required for successful *d*STORM reconstructions with home-built setups. Depending on the fluorophore, a certain minimum laser power is needed to enhance the photoswitching rate of the fluorophores in order to adjust the blinking rate in one frame^21^,^23^,^29^. With distinct cellular structures such as microtubules, *d*STORM imaging can be achieved more readily because the labeling density is restricted to just a single optical plane (for very flat cells). Accordingly, our resulting images showed highly resolved tubulin structures (Figs 1 and 3) that were well defined in isolated filaments but slightly less so at points where crossing filaments overlap.

In order to investigate structures with negative image contrast by *d*STORM, such as fenestrations in the cellular plasma membrane, on the other hand, the sample has to be stained with a dye coverage that is as uniform and dense as possible to avoid potential artifacts such as non-existing holes that might appear due to spotty membrane staining. We previously showed that CellMask Deep Red (CMDR) is a useful membrane stain for this application^16^ (to the best of our knowledge there are no specific markers for LSEC fenestrations). Imaging a densely labeled cellular membrane can be compared to imaging an extended 2D structure. Each fluorophore has multiple neighboring fluorophores, increasing the likelihood of overlapping single molecule emissions in single frames. Unless multi-emitter fitting algorithms are employed to analyze such data, these overlaps between blinking events of single molecules need to be avoided by appropriately adjusting the laser exposure power. Although CMDR is an excellent plasma membrane stain, well suited for fixed and live cell experiments, it leads to high background contributions from regions outside the cell. In this paper we show that Vybrant DiD labeling of membranes is better suited for *d*STORM acquisitions (Suppl. Fig. S1). With this fluorophore, 50% of the full laser power of our 642 nm diode laser was sufficient to induce appropriate blinking rates of the fluorophore. If required, Vybrant DiD can be activated using 405 nm or 488 nm in order to adjust the density of blinking emitters in a single frame. DiD appears to be highly specific for cell membranes and thus shows extremely low background contributions. In the 3D-SIM vs. *d*STORM comparison of Vybrant DiD stained LSECs the 3D-SIM reconstructed super-resolution image clearly depicts fenestrations arranged in sieve plates. Only the area in the immediate vicinity of the nucleus lacks fenestrations (see Fig. 2A,C). A clear resolution gain can be observed in the corresponding *d*STORM image. Although the cell was illuminated at a slightly inclined angle in HiLo mode, the *d*STORM reconstruction (which does not use cylindrical lens-induced astigmatism to enable 3D reconstruction) produces a pure 2D image. Here, Vybrant DiD labeling shows excellent membrane specificity and appears to be devoid of background contributions (Fig. 2B,D).

Ideally a comparative analysis of 3D-SIM and *d*STORM would use a switching buffer system that is suitable for both super-resolution methods, especially since the chemical environment is crucial for facilitating successful *d*STORM reconstructions. Most measurements are therefore conducted using Oxygen Scavenging System (OSS) buffers. For 3D-SIM imaging, Vectashield is the preferred mounting medium mostly due to its well suited index of refraction that enables aberration-reduced imaging of cells. On the other hand it turns out that Vectashield also works well with several different dyes (e.g. Alexa647, DiD, etc.) to enable single molecule based localization microscopy^24^. Another advantage of using Vectashield is that the mounted samples are more photostable than with aqueous OSS-buffers. Even weeks after mounting, DiD showed a photoswitching behavior comparable to that of freshly prepared samples (data not shown).

The combination of two super-resolution optical modalities on the same setup fuses the great practical abilities of 3D-SIM, which makes it so attractive for rapid multi-color bioimaging, with the significantly higher spatial resolution of *d*STORM. 3D-SIM enables fast (0.5 s for a 0.8 μm stack) three-dimensional measurements with a lateral spatial resolution of about 100 nm in up to 4 different color channels. It allows for rapid scanning of large sample areas with subsequent image stitching in software to compose large super-resolved overview images. After pre-scanning samples with SIM, *d*STORM imaging of selected ROIs permits us to revisit select sample locations with even higher spatial resolution. In our commercial system, the built-in autofocus unit supports the stability in the vertical direction to avoid drift, while drift in the lateral direction could be corrected afterwards in the reconstruction software^30^. Remarkably, multi-color staining of LSECs clearly shows that microtubuli appear to surround and separate sieve plates (see Fig. 3B), while actin filaments surround each fenestrae within a sieve plate (see Fig. 4). This further confirms the hypothesis that the cytoskeleton is of great importance for either stabilizing or even defining fenestrations in LSECs^10^. From the *d*STORM/3D-SIM overlay image and corresponding line sections shown in Fig. 4D–G it can be seen that actin filaments appear to form a scaffold structure that surrounds and possibly supports the fenestrations. Microtubuli create borders for fenestrations that assemble in sieve plates, while actin appears to stabilize the membrane which is extremely thin in these areas.

## Conclusions

We have demonstrated that the combined use of two super-resolution optical microscopy modalities, 3D-SIM and *d*STORM, enables us to observe the sub-diffraction sized LSEC fenestrations with great detail down to 20 nm. This allows us to directly compare the ultrastructure of the cytoskeleton and the plasma membrane with the highest possible optical resolution. Current protocols do not lead to 100% purity in LSECs isolation. Other liver cell types are typically contaminating the LSEC cultures. Imaging cells by 3D-SIM is significantly faster in acquisition and leaner in data volume than *d*STORM. Therefore, rapid identification of LSECs by 3D-SIM according to the presence and morphology of fenestrations could be a useful means for finding and confirming the identity of LSECs, especially for future live cell studies. The vast rapid survey of plated cells by 3D-SIM can then be followed by subsequent *d*STORM analysis at even higher spatial resolution. Interestingly, this combination also enables us to directly compare cellular structures that were imaged by different microscopy methods, where, under certain circumstances a lower-resolving, but faster, three-dimensional, and multicolor imaging method, such as 3D-SIM can provide added value for the analysis of the connection between different cellular constituents.

3D-SIM is capable of fast acquisitions that are suited for live cell imaging (up to <0.5 s per image). Live observations of the dynamics of actin / tubulin filaments, fenestrations and entire sieve plates will significantly contribute to our understanding of their respective functions and the correlation between them^31^. By combining two super-resolution imaging modalities it will e.g. be possible to first image the dynamics of living LSECs in multi-color 3D-SIM mode, followed by *in-situ* fixation of the sample and subsequent recording of the same cells at even higher spatial resolution by *d*STORM. This will provide us with an initial approach allowing us to overcome one of the current limitations of super-resolution microscopy, i.e. the combination of rapid live cell imaging of cellular processes with the highest spatial resolution possible.

## Materials and Methods

### Materials

Reagents included Type 1 A Collagenase (Sigma Chemical, St. Louis, MO #C2674,) RPMI (Gibco Invitrogen #11875-093), CellMask Deep Red Plasma Membrane Stain, Invitrogen, #C10046 Mouse monoclonal Anti-β-Tubulin antibody, SIGMA, T8328 Alexa Fluor® 647 F(ab’)2 Fragment of Goat Anti-Mouse IgG (H + L), Invitrogen, A-21237, Lot# 1094366 CellMask Orange 5 mg/ml, Invitrogen, C10045, Lot# 1159930 Alexa Fluor 488 – phalloidin 300 u in 1.5 ml Methanol, Invitrogen, A12379, Lot# 1120408 Vybrant DiD cell-labeling solution 1 mM, Invitrogen, V22887, Lot# 1046290 VECTASHIELD Mounting Medium with DAPI, Vector Laboratories, H-1200, Collagen and Fibronectin were purchased from Sigma Chemical.

### Isolation and culture of LSECs

Sprague Dawley male rats (Scanbur BK, Sollentuna, Sweden) were kept under standard conditions and fed standard chow ad libitum (Scanbur, Nittedal, Norway). The experimental protocols were approved by the Norwegian National Animal Research Authority (NARA) in accordance with the Norwegian Animal Experimental and Scientific Purposes Act of 1986. All experiments were performed in accordance with relevant approved guidelines and regulations. The rats (body wt 150–300 g) were anesthetized with a mixture of medetomidin (Domitor vet, Orion, Turku, Finland) and ketamine (Ketalar, Pfizer, New York, NY) and LSECs were isolated and purified as described^32^ and plated on fibronectin coated #1.5 coverslips for 3 h in RPMI-1640. The LSECs were then fixed with 4% paraformaldehyde (PFA) in phosphate buffered saline (PBS) and 0.02 M sucrose, pH 7.2 for 15 min, and stored under PBS. Following fixation the cells were prepared for visualization using 3D-SIM and *d*STORM.

### Fluorescent staining of LSECs and imaging buffers

The fixed samples were washed with PBS. For Figs 1, 3 and 4 the LSECs were permeabilised for 90 s with 0.5% Triton-X100 and washed 2–3 times with PBS. Non-specific binding sites were blocked by incubating the cells for 90 min with 5% BSA in PBS at 4 °C. The cells were incubated with the primary antibody (mouse anti beta-tubulin, 1:400) for 2 h at RT, then washed 3 times for 10 min with 0.1% PBS Tween-20. The secondary antibody (anti mouse AlexaFluor647, 1:400) was applied overnight at 4 °C. The staining solution was removed and washing steps with 0.1% PBS Tween-20 (3 times for 10 min) and PBS followed. The phalloidin AlexaFluor488 (1:40) and CellMask Orange (1:2000) were applied together for 20 min at 4 °C. Cells were washed with PBS and post-fixed with 4% PFA for 10 min, followed by a PBS washing step.

The LSECs in Fig. 4 were incubated with phalloidin AlexaFluor488 (1:40) and Vybrant DiD (1:100) overnight at 4 °C. All samples were mounted in Vectashield containing DAPI and sealed with nail polish. For Suppl. Figure S1, LSECs were stained with CellMask Deep Red (1:2000), or Vybrant DiD (1:200) for 20 min at RT, respectively. For Fig. 2 LSECs were stained with Vybrant DiD (1:200) for 20 min at RT. After removing the dye and rinsing each sample 3 times with PBS, these samples were mounted with a reservoir of an oxygen scavenging system including MEA^22^.

### Super resolution imaging of LSECs

3D-SIM images of LSECs were acquired on a commercial structured illumination microscope (DeltaVision|OMXv4.0 BLAZE, GE Healthcare) and reconstructed using the SoftWorX package from GE Healthcare. To achieve even higher spatial resolution of microtubules and fenestrations we used single molecule localization microscopy of conventional fluorophores (direct Stochastic Optical Reconstruction Microscopy - *d*STORM) on the same setup by changing the illumination mode from structured illumination to ring-TIRF (total internal reflection fluorescence excitation), albeit at an angle just below the critical angle to obtain a highly inclined laminated optical sheet (HiLo) illumination mode.

To enable their controlled blinking, the AlexaFluor647 and DiD molecules were excited with a 642 nm laser and recovered from the dark state either with a weak 405 nm or 488 nm laser. The 642 nm laser power was set for Alexa647 at 100% and for DiD at 50% such that the density of the fluorophores in the fluorescent state is as high as possible while still being well separated to enable single molecule localization. Typical excitation laser power densities were approximately 0.25–0.5 kW/cm^2^. The acquisition time was 10–30 ms per frame and 10,000–15,000 frames were recorded using a sCMOS camera (total time ~10–15 min). Raw data were preprocessed to reduce background signal and single pixel characteristics intrinsic to sCMOS cameras by subtracting the average of the image stack from each individual frame.

For the final reconstruction of a *d*STORM image at least 10,000 frames are analyzed by the open source reconstruction software rapidSTORM^30^,^33^. Images were corrected for sample drift by assuming linear drift using the built-in drift correction of rapidSTORM.

## Additional Information

**How to cite this article**: Mönkemöller, V. *et al.* Multimodal super-resolution optical microscopy visualizes the close connection between membrane and the cytoskeleton in liver sinusoidal endothelial cell fenestrations. *Sci. Rep.* **5**, 16279; doi: 10.1038/srep16279 (2015).

## Supplementary Material

Supplementary Movie 1

Supplementary Information

## References

[b1] Betzig E. Proposed Method for Molecular Optical Imaging. Opt. Lett. 20, 237–239 (1995). 19859146 10.1364/ol.20.000237

[b2] Betzig E. *et al.* Imaging intracellular fluorescent proteins at nanometer resolution. Science 313, 1642–1645 (2006). 16902090 10.1126/science.1127344

[b3] Gustafsson M. G. L., Agard D. A. & Sedat J. W. Doubling the lateral resolution of wide-field fluorescence microscopy using structured illumination. Proc. SPIE 3919, 141–150 (2000).

[b4] Gustafsson M. G. L. *et al.* Three-dimensional resolution doubling in wide-field fluorescence microscopy by structured illumination. Biophys. J. 94, 4957–4970 (2008). 18326650 10.1529/biophysj.107.120345 PMC2397368

[b5] Hell S. W. Far-field optical nanoscopy. Science 316, 1153–1158 (2007). 17525330 10.1126/science.1137395

[b6] Hell S. W. & Wichmann J. Breaking the Diffraction Resolution Limit by Stimulated-Emission - Stimulated-Emission-Depletion Fluorescence Microscopy. Opt. Lett. 19, 780–782 (1994). 19844443 10.1364/ol.19.000780

[b7] Shao L., Kner P., Rego E. H. & Gustafsson M. G. L. Super-resolution 3D microscopy of live whole cells using structured illumination. Nat. Methods 8, 1044–1046 (2011). 22002026 10.1038/nmeth.1734

[b8] Kner P., Chhun B. B., Griffis E. R., Winoto L. & Gustafsson M. G. L. Super-resolution video microscopy of live cells by structured illumination. Nat. Methods 6, 339–342 (2009). 19404253 10.1038/nmeth.1324 PMC2895555

[b9] Fiolka R., Shao L., Rego E. H., Davidson M. W. & Gustafsson M. G. Time-lapse two-color 3D imaging of live cells with doubled resolution using structured illumination. Proc. Natl. Acad. Sci. USA 109, 5311–5315 (2012). 22431626 10.1073/pnas.1119262109 PMC3325651

[b10] Braet F. & Wisse E. Structural and functional aspects of liver sinusoidal endothelial cell fenestrae: a review. Comp. Hepatol. 1, 10.1186/1476-5926-1-1 (2002). PMC131011 12437787

[b11] Smedsrod B., Pertoft H., Eggertsen G. & Sundstrom C. Functional and morphological characterization of cultures of Kupffer cells and liver endothelial cells prepared by means of density separation in Percoll, and selective substrate adherence. Cell Tissue Res. 241, 639–649 (1985). 2992796 10.1007/BF00214586

[b12] Wisse E. An electron microscopic study of the fenestrated endothelial lining of rat liver sinusoids. J. Ultrastruct. Res. 31, 125–150 (1970). 5442603 10.1016/s0022-5320(70)90150-4

[b13] Braet F. *et al.* Structure and dynamics of the fenestrae-associated cytoskeleton of rat liver sinusoidal endothelial cells. Hepatology 21, 180–189 (1995). 7806153

[b14] Cogger V. C. *et al.* Three-dimensional structured illumination microscopy of liver sinusoidal endothelial cell fenestrations. J. Struct. Biol. 171, 382–388 (2010). 20570732 10.1016/j.jsb.2010.06.001 PMC3043550

[b15] Svistounov D. *et al.* The Relationship between Fenestrations, Sieve Plates and Rafts in Liver Sinusoidal Endothelial Cells. Plos One 7, e46134 (2012). 23029409 10.1371/journal.pone.0046134 PMC3454341

[b16] Mönkemoller V. *et al.* Imaging fenestrations in liver sinusoidal endothelial cells by optical localization microscopy. Phys. Chem. Chem. Phys. 16, 12576–12581 (2014). 24830784 10.1039/c4cp01574f

[b17] Matsuda A. *et al.* Condensed Mitotic Chromosome Structure at Nanometer Resolution Using PALM and EGFP- Histones. Plos One 5, e12768 (2010). 20856676 10.1371/journal.pone.0012768 PMC2939896

[b18] Rossberger S. *et al.* Combination of structured illumination and single molecule localization microscopy in one setup. J. Opt. 15, 094003 (2013).

[b19] Hamel V. *et al.* Correlative multicolor 3D SIM and STORM microscopy. Biomed. Opt. Express 5, 3326–3336 (2014). 25360353 10.1364/BOE.5.003326 PMC4206305

[b20] Heilemann M., van de Linde S., Mukherjee A. & Sauer M. Super-resolution imaging with small organic fluorophores. Angew. Chem. Int. Ed. 48, 6903–6908 (2009). 10.1002/anie.200902073 19670280

[b21] Heilemann M. *et al.* Subdiffraction-resolution fluorescence imaging with conventional fluorescent probes. Angew. Chem. Int. Ed. 47, 6172–6176 (2008). 10.1002/anie.200802376 18646237

[b22] van de Linde S. *et al.* Direct stochastic optical reconstruction microscopy with standard fluorescent probes. Nat. Protoc. 6, 991–1009 (2011). 21720313 10.1038/nprot.2011.336

[b23] van de Linde S., Sauer M. & Heilemann M. Subdiffraction-resolution fluorescence imaging of proteins in the mitochondrial inner membrane with photoswitchable fluorophores. J. Struct. Biol. 164, 250–254 (2008). 18790061 10.1016/j.jsb.2008.08.002

[b24] Olivier N., Keller D., Rajan V. S., Gonczy P. & Manley S. Simple buffers for 3D STORM microscopy. Biomed. Opt. Express 4, 885–899 (2013). 23761850 10.1364/BOE.4.000885 PMC3675867

[b25] Tokunaga M., Imamoto N. & Sakata-Sogawa K. Highly inclined thin illumination enables clear single-molecule imaging in cells. Nat. Methods 5, 159–161 (2008). 18176568 10.1038/nmeth1171

[b26] Schindelin J. *et al.* Fiji–an Open Source platform for biological image analysis. Nat. Methods 9, 676–682 (2012). 22743772 10.1038/nmeth.2019 PMC3855844

[b27] Vaughan J. C., Jia S. & Zhuang X. W. Ultrabright photoactivatable fluorophores created by reductive caging. Nat. Methods 9, 1181–1184 (2012). 23103881 10.1038/nmeth.2214 PMC3561463

[b28] Endesfelder U. & Heilemann M. Art and artifacts in single-molecule localization microscopy: beyond attractive images. Nat. Methods 11, 235–238 (2014). 24577272 10.1038/nmeth.2852

[b29] van de Linde S. *et al.* Multicolor photoswitching microscopy for subdiffraction-resolution fluorescence imaging. Photochem. Photobiol. Sci. 8, 465–469 (2009). 19337659 10.1039/b822533h

[b30] Wolter S. *et al.* rapidSTORM: accurate, fast open-source software for localization microscopy. Nat. Methods 9, 1040–1041 (2012). 23132113 10.1038/nmeth.2224

[b31] Li D. *et al.* Extended resolution structured illumination imaging of endocytic and cytoskeletal dynamics. Science 349, aab3500 (2015). 26315442 10.1126/science.aab3500 PMC4659358

[b32] Smedsrod B. & Pertoft H. Preparation of pure hepatocytes and reticuloendothelial cells in high yield from a single rat liver by means of Percoll centrifugation and selective adherence. J. Leukoc. Biol. 38, 213–230 (1985). 2993459 10.1002/jlb.38.2.213

[b33] Wolter S., Endesfelder U., van de Linde S., Heilemann M. & Sauer M. Measuring localization performance of super-resolution algorithms on very active samples. Opt. Express 19, 7020–7033 (2011). 21503016 10.1364/OE.19.007020

