# Supplementary material for: Multimodal super-resolution optical microscopy visualizes the close connection between membrane and the cytoskeleton in liver sinusoidal endothelial cell fenestrations
Source: Sci Rep. 2015 Nov 9;5:16279. doi: 10.1038/srep16279 (PMC4637861; doi:10.1038/srep16279)
Supplement: Supplementary Information [file srep16279-s2.doc]

**Supplementary Information**

**Multimodal super-resolution optical microscopy visualizes the close connection between membrane and the cytoskeleton in liver sinusoidal endothelial cell fenestrations**

Viola Mönkemöllera,*, Cristina Øieb,*, Wolfgang Hübnera, Thomas Husera,c, and Peter McCourtb

aBiomolecular Photonics, Department of Physics, Bielefeld University, Universitätsstr. 25, 33615 Bielefeld, Germany
bFaculty of Health Sciences, Department of Medical Biology, Vascular Biology Research Group, University of Tromsø, The Arctic University of Norway Tromsø, Norway

cDepartment of Internal Medicine, and NSF Center for Biophotonics, University of California, Davis, 2700 Stockton Blvd., Ste. 1400, Sacramento, CA 95817, USA

Supplementary Note:

We evaluated the ability of our commercial 3D-SIM platform to enable *d*STORM imaging of plasma membrane stains. The result is shown in Supplementary Figure 1, where we compared the performance of two different plasma membrane stains: CellMask Deep Red (CMDR, Fig. S1A), and Vybrant DiD (Fig. S1B). As is apparent from these images, both dyes stain sieve plates and fenestrations quite well. For CMDR a higher laser power than what is available with our commercial 3D-SIM platform is needed for successful *d*STORM imaging, resulting in less than ideal *d*STORM measurements (see Fig. S1A), which explains the "granularity" of the *d*STORM images compared to our previously published results. Even though CMDR is an excellent plasma membrane stain, well suited for fixed and live cell experiments, it leads to high background contributions from regions outside the cell in the *d*STORM reconstructions due to nonspecific binding of the probe to the substrate (Fig. S1A). Recording in TIRF mode only enhances this effect. CMDR appears to have a considerable affinity to fibronectin or collagen coated glass slides, limiting its use for *d*STORM imaging in TIRF mode. Vybrant DiD labeling of membranes, on the other hand, is very well suited for *d*STORM acquisitions (Fig. S1B).


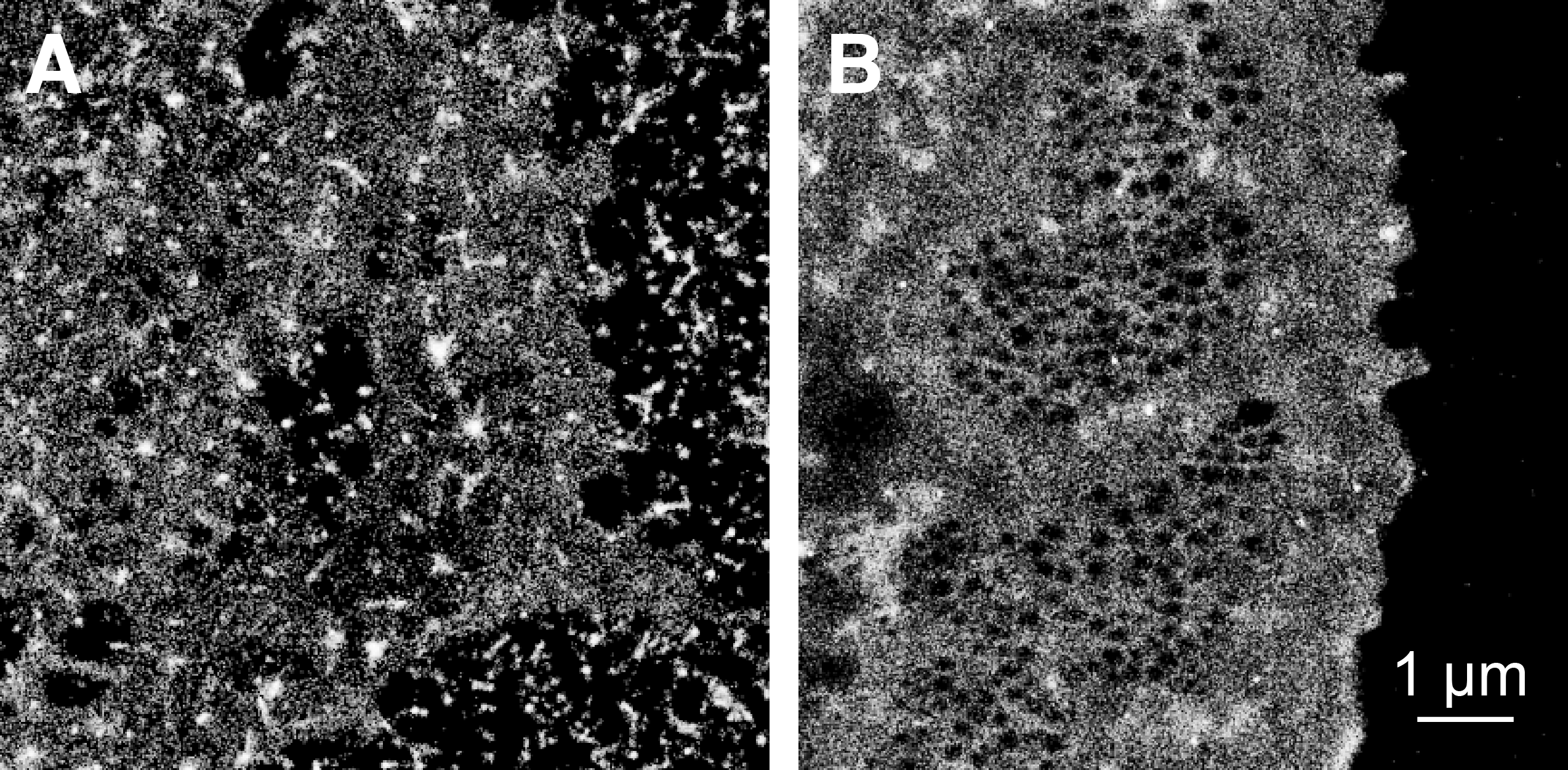


**Supplementary Figure S1: Comparing the effects of different membrane stains in *d*STORM measurements**

(A) *d*STORM image of a fixed rat LSEC stained with CellMask Deep Red. Note the significant unspecific binding of the stain to the substrate which makes it nearly impossible to discern cell and background. (B) *d*STORM image of another fixed rat LSEC stained with Vybrant DiD. Note the reduced unspecific binding to the background.


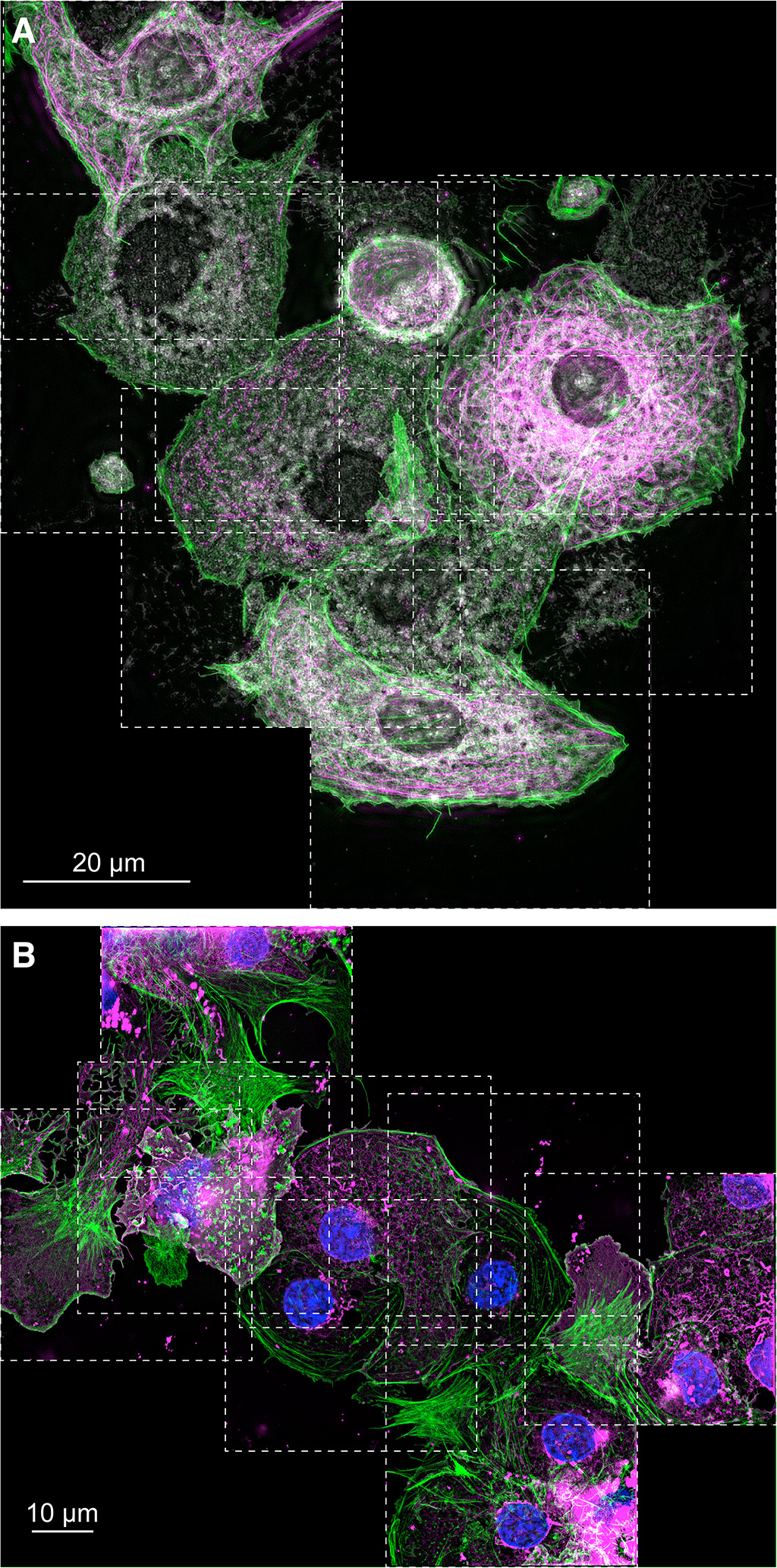


**Supplementary Figure S2: Stitching of large 3D-SIM images**

(A) Combined stitch of the 7 original 3D-SIM images outlined by white dashed boxes into a single overview image (compare to Fig. 3A). (B) Combined stitch of the 8 original 3D-SIM images outlined by white dashed boxes into a single overview image (compare to Fig. 4A).

**Supplementary Movie 1: Animated flyover and 3D view of Figure 4A**

Flyover across the stitched image shown in Fig. 4A, where in a zoomed part of the figure the original 3D-SIM data are used to demonstrate the three-dimensional nature of structured illumination microsopy. Nuclei are shown in blue, actin cytoskeleton in green, and plasma membrane in red. (Flyover and 3D animation generated in OsiriX)
